# Current Knowledge About Aprocitentan in Hypertension

**DOI:** 10.3390/ijms262311431

**Published:** 2025-11-26

**Authors:** Emilie Mathilde Bank-Mikkelsen, Daniela Grimm, Markus Wehland

**Affiliations:** 1 Department of Biomedicine, Aarhus University, 8000 Aarhus, Denmark; au729370@uni.au.dk (E.M.B.-M.); dgg@biomed.au.dk; 2 Department of Microgravity and Translational Regenerative Medicine, Otto von Guericke University, 39106 Magdeburg, Germany

**Keywords:** aprocitentan, dual endothelin receptor antagonist, hypertension

## Abstract

Hypertension (HT) is the leading contributor to the global burden of disease and overall mortality and is expected to increase due to such factors as increased life expectancy and rising obesity rates. Although HT significantly contributes to cardiovascular disease, it is also considered one of the most modifiable risk factors. Aprocitentan (ACT) is a newly developed orally administered dual endothelin receptor antagonist. This review aims to give an overview of the current knowledge regarding ACT in HT, focusing on its pharmacological mechanisms and therapeutic potential. We conducted a search in the PubMed and Clinicaltrials.gov databases using the search terms “hypertension”, “aprocitentan”, high blood pressure” and “cardiovascular disease”, as well as all their permutations. Both human and animal studies have demonstrated significant blood pressure reductions within 14 days of administration, with 25 mg identified as the most effective dose and no severe adverse effects. Moreover, ACT was compatible with other antihypertensive agents, demonstrating synergistic or additive effects in some cases. Since HT is frequently associated with comorbidities and ACT targets a different pathway than the existing antihypertensive drugs, ACT may play a pivotal role in the management of resistant hypertension.

## 1. Introduction

Hypertension (HT) is the leading contributor to the global burden of disease and overall mortality [1]. The prevalence of HT increases with age, with estimates suggesting that individuals under the age of 60 have a 27% risk of HT, whereas those over 80 years of age have a 74% risk [2]. According to the WHO, 1.28 billion adults worldwide were affected by HT in 2023, with the highest burden observed in low- and middle-income countries. Uncontrolled HT can lead to cardiovascular diseases ( CVDs) like heart failure, myocardial infarction, and stroke. Beyond its cardiovascular implications, uncontrolled HT is also a contributor to renal impairment, including kidney damage or failure [3]. Due to its high prevalence as well as its association with serious complications such as CVD and kidney disease, HT represents a significant global health concern.

The burden of HT is expected to increase in parallel with the rising life expectancy, with the proportion of people aged 65 and older estimated to reach 22% of the population in 2033. In a global study from 2021 [4], it was found that between 1990 and 2019, the number of cases of HT in persons between the age of 30–79 years increased from 331 million to 626 million for women and from 317 million to 652 million for men, respectively. Furthermore, the costs of HT treatment are projected to rise substantially [5,6]. While standard treatment drugs are well-established and proven to be effective, some patients are non-responsive to this regimen. Therefore, it is important to develop more drugs targeting different pathways in order to provide a treatment option for this patient group. This review aims to give a comprehensive overview of the current knowledge on clinical trials with aprocitentan, a newly developed orally administered dual endothelin receptor antagonist.

## 2. Hypertension

**Figure 1:**
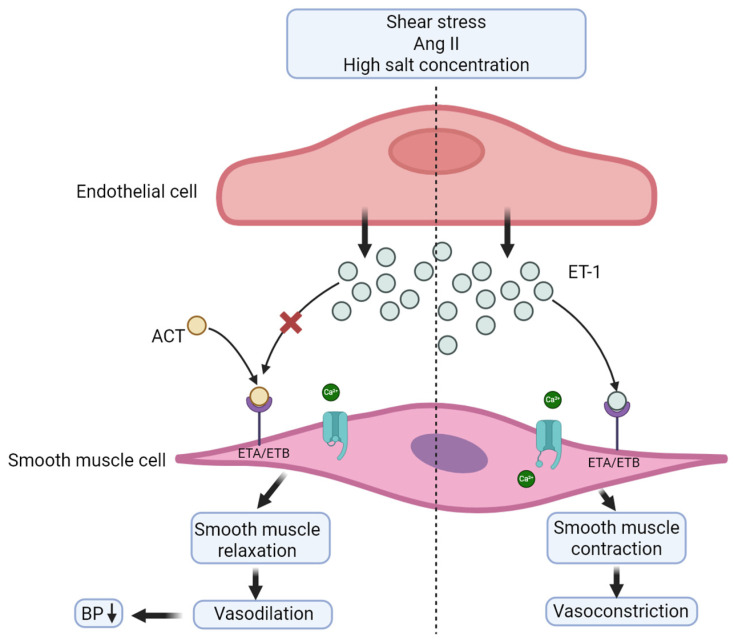
Effects of aprocitentan and ET-1. The figure illustrates the effects of aprocitentan (ACT) and endothelin 1 (ET-1). When the endothelial cell is stimulated, it releases ET-1 (blue particles). ET-1 binds to its receptor (ETA and/or ETB), which results in a depolarization of the membrane and thereby opening of the voltage gated L-type calcium channels (blue channel), resulting in an influx of calcium (green particle). This leads to smooth muscle contraction and thereby vasoconstriction. Aprocitentan/ACT (orange particle) is an ETA/ETB antagonist, if present it will block the ET-1 receptor, resulting in smooth muscle relaxation, leading to vasodilation and subsequently a reduction in blood pressure (BP). The figure is based on information from [7,8,9] and was Created in BioRender. Wehland, M. (2025) https://BioRender.com/f0s5ou8, last accessed on 17 May 2025.

One key modulator of vascular tone is endothelin (ET), which is released as a response to several stimuli, some of which are angiotensin II (Ang II) and shear stress [7]. ET exists in three isoforms (ET-1 to ET-3). ET-1, a potent vasoconstrictor secreted by endothelial cells, activates endothelin receptors ETA and ETB2, located on vascular smooth muscle cells. Upon binding, ET-1 induces calcium (Ca^2+^) influx through membrane channels, thereby facilitating smooth muscle contraction. Dysregulation in the secretion of ET-1, leading to an increased expression, has been implicated to play a part in the pathogenesis of HT [8,9]. A simplified schematic overview of the effects of ET-1 is depicted in Figure 1.

### Forms of Hypertension

There are different types of HT, essential or primary and secondary HT. For essential HT there are no identifiable causes for the elevated BP, and it develops gradually over several years [10]. Secondary HT, on the other hand, has an underlying and potentially reversible cause, but only makes up a small fraction of hypertensive cases [11].

Resistant or treatment-resistant HT is defined as persistently elevated BP exceeding 190/40 mmHg despite the concurrent use of three antihypertensive medications at optimal dosage, including a diuretic, with the BP remaining above the therapeutic target. To confirm the diagnosis of treatment-resistant HT, causes of secondary HT are required to be excluded [12].

## 3. Aprocitentan

Aprocitentan (ACT), ACT-132577, is a new antihypertensive drug developed by Idorsia Pharmaceuticals (Allschwill, Switzerland) as a potential treatment for resistant hypertension. The main purpose of ACT is to target the endothelin receptors and block their effects [13]. ACT is the active metabolite of macitentan, a drug used for treating pulmonary arterial hypertension [14].

### 3.1. Mechanism of Action

ACT is an orally administered dual ETA and ETB receptor antagonist (ERA), which inhibits the binding of ET-1 to its receptor by selectively binding to them itself. It exhibits greater selectivity for the receptor, thereby effectively blocking ET-1-mediated pathways. By binding to the ET receptors, aprocitentan inhibits calcium influx, thereby reducing the contraction of vascular smooth muscle, leading to vasodilation and a subsequent reduction in BP (31). ACT has a selectivity ratio of 1:16 for the ETA and ETB2 receptor, which supports its efficacy as an ET-1 antagonist [15]. Studies have observed significant changes in BP within 14 days of administering ACT [14].

The production of ET-1 is stimulated by several factors, among them is high salt intake, making it a relevant factor in hypertensive patients consuming a high-sodium diet. Such patients exhibit elevated ET-1 levels, which contribute to the pathophysiology of HT [16]. Furthermore, ET-1 is recognized as the most potent endogenous vasoconstrictor, strongly implicating it in the pathogenesis of HT [17]. In a study conducted by Trensz et al., inhibition of both ETA and ETB receptors resulted in a dose-dependent reduction in BP in rats. Moreover, plasma concentrations of ET-1 in rats treated with ACT were significantly increased, further demonstrating its effectiveness. ACT was found to be more effective in lowering BP in deoxycorticosterone acetate (DOCA)-salt rats with induced HT compared to spontaneously hypertensive rats (SHRs). Moreover, it showed a decrease in cardiomyopathy and renal vascular resistance without affecting HR in the DOCA-salt rats [16]. ACT targets a different pathway than the existing first-in-line antihypertensive agents and is, therefore, expected to provide additive BP-lowering effects when used in combination with other antihypertensive drugs, such as RAAS blockers [16].

In contrast to some other antihypertensive drugs, ACT is not associated with hepatotoxicity in rodent studies. Furthermore, it appears to pose a lower risk of fluid retention and vascular leakage [18]. Figure 1 shows a schematic overview of the effects of ACT on the vascular smooth muscle cells.

### 3.2. Pharmacokinetics

The pharmacokinetics of ACT have been investigated in clinical trials. Table 1 provides an overview of the plasma pharmacokinetics of ACT following the administration of various doses.

According to Sidharta et al., the first study assessing the tolerability of single and multiple doses in humans, ACT is highly bound to plasma proteins and is eliminated in feces and urine. The study finds that ACT reached its maximum plasma concentration (C_max_) approximately 3–4 h after administration, with an elimination half-life (t_½_) of approximately 41–48 h, fitting a once-daily dose. Furthermore, it was observed that adult females exhibited a longer systemic exposure to ACT compared to males under both single- and multiple-dose steady-state conditions. Additionally, the elimination half-life was 1.5 h shorter in the elderly, suggesting accelerated metabolism of ACT in this subgroup. However, this difference was considered clinically insignificant. Moreover, the presence of food did not significantly affect the tolerability of ACT [19].

Another study, by Fontes et al. on 20 healthy males and females of Japanese and Caucasian descent found approximately the same C_max_ and t_½_ as mentioned above, with a slightly higher t_½_ for Japanese subjects at 49.1 h, although this was not deemed relevant and it was concluded that there was no significant difference between ethnic groups [20].

### 3.3. Interaction

Investigations of the interactions of ACT with different drugs are essential since resistant HT is frequently associated with comorbidities, and patients therefore require multiple medications [21]. ACT is a newly developed antihypertensive drug and is therefore still being tested, which limits the current knowledge regarding the interactions between ACT and other drugs. However, ACT has been tested in combination with both valsartan and enalapril, an angiotensin-II type-1 receptor blocker and ACE inhibitor, respectively. According to Trensz et al., ACT exhibited greater pharmacological activity than valsartan alone, and the combined administration of ACT and valsartan produced a synergistic effect, yielding a larger reduction in BP compared to monotherapy. The combination of ACT and enalapril demonstrated a synergistic effect in DOCA-salt rats, whereas in SHRs, the effect was only additive [16]. Furthermore, the interaction potential of ACT has been evaluated in a clinical trial with rosuvastatin, a breast cancer resistance protein (BCRP) transporter substrate (NCT03245229) [22], and with combined hormonal contraceptives (NCT06799884) [23]. No results have yet been posted for the contraceptive study, as it was only recently completed (21 February 2025). However, the study assessing the effects of ACT on rosuvastatin found that ACT increased the C_max_ but slightly lowered the t_½_ of rosuvastatin. Overall, it was deemed that ACT did not affect the PK and tolerability of rosuvastatin in a clinically significant way [22].

### 3.4. Indication

ACT is a drug that can be used in the treatment of HT. As previously mentioned, ACT is an ERA, which inhibits the effects of ET-1. Inhibition of the ET system has been shown to reduce BP; therefore, elevated BP represents a clinical indication for the use of ACT, making it a favorable option for hypertensive patients [24]. Furthermore, ACT has demonstrated additive and synergistic effects when combined with other antihypertensive agents, enhancing cardiovascular protection in patients with resistant HT [14].

## 4. Clinical Trials

Table 2 provides an overview of recent clinical trials regarding ACT in relation to HT and the safety and tolerability of ACT.

The study by Verweij et al. (NCT02603809) found that a clinically relevant decrease in automatically measured office BP occurred within 2 weeks in the ACT 10, 25, and 50 mg groups. The reduction was maintained until the end of the study. Following withdrawal, the BP returned to placebo levels, suggesting the absence of a rebound effect. The maximum effects were observed at a 25 mg ACT dose but were statistically estimated to be reached at 31 mg [18]. The study by Fontes et al. (NCT03586570) reported no significant differences in plasma concentrations between treatment groups. Moreover, plasma concentrations declined slowly, as indicated by the mean half-time. Finally, no clinically relevant differences in PK were reported between sexes [20]. The study by Schlaich et al. (NCT03541174) was a phase 3 trial. Part 1, a double-blinded, randomized treatment part (4 weeks), involved patients continuing their individual background therapy, after which they received ACT 12.5 mg, ACT 25 mg, or placebo. The least square mean change in office SBP was −15.3 mmHg for ACT 12.3 mg, −15.2 mmHg for ACT 25 mg, and −11.5 mmHg for placebo, demonstrating a greater decrease in the ACT groups. Office DBP also decreased from both ACT doses. Part 2, a single (patient)-blinded active-treatment part (32 weeks), administered ACT 25 mg to all patients. BP levels were maintained in patients previously receiving ACT, while a decrease was observed in those previously on placebo. Part 3, a double-blinded, placebo-controlled, randomized withdrawal part (12 weeks), re-randomized participants to receive either ACT 25 mg or placebo. A significant increase in BP was observed in patients receiving placebo, confirming the sustained efficacy of ACT in lowering BP. Furthermore, the study identified potential organ-protective effects in patients with kidney disease. The addition of ACT adequately treated patients with resistant hypertension produced a clinically meaningful reduction in BP, as measured by standardized automated office BP and 24 h ambulatory BP, compared to the placebo group, after 4 weeks of treatment. A pronounced reduction in nocturnal BP was also observed, which is considered a superior predictor of cardiovascular mortality, emphasizing the clinical relevance of this finding [25]. The study by Fontes et al. (NCT04252495) observed similar plasma concentration-time profiles of ACT between moderate hepatic impaired and healthy subjects, after 14 days of treatment. Although the apparent clearance was slightly reduced for participants with moderate hepatic impairment, this resulted in a modestly prolonged exposure [26].

A meta-analysis conducted by Wang et al. found that monotherapy with standard doses of ACEIs, ARBs, BARBs, CCBs or diuretics reduced the mean systolic blood pressure by 6.8, 8.5, 8.9, 9.5, and 10.8 mmHg, respectively. The mean reduction for all monotherapies taken together was 8.7 mmHg. They also screened 0.5× and 2× doses and found that each doubling conferred an additional reduction by 2.5 (1.4–3.7) mmHg [27]. In comparison to that, the dose-finding study by Verweij et al. in patients with essential HT receiving ACT monotherapy found that doses of 10 and 25 mg ACT lowered the mean systolic blood pressure by 7.05 and 9.0 mmHg, respectively [18], which lies within the range of the other antihypertensive drugs. Furthermore, it was shown, that doses of 12.5 and 25 mg of ACT on top of standardized baseline therapy consisting of a combination of amlodipine, valsartan, and hydrochlorothiazide at maximally tolerated doses resulted in an additional reduction in office systolic blood pressure by 3.8 and 3.7 mmHg, respectively [25].

Overall, all trials investigating ACT in relation to HT have demonstrated that it effectively lowers BP. In addition, ACT exhibits an additive or synergistic effect when combined with other antihypertensive agents. None of the studies reported any severe AEs, suggesting that ACT is well tolerated across all doses. Furthermore, a dose of 25 mg has consistently been indicated as the most optimal [18,20,25,26].

Currently, no studies on ACT in relation to HT are recruiting participants. A phase 3 study (Inspire-CKD, NCT04162366) was withdrawn due to business decisions, and no results were reported [28].

## 5. Discussion

### 5.1. Safety and Adverse Effects

Overall, ACT has been reported to be well tolerated, with minimal adverse effects (AEs). In the study by Fontes et al. (NCT04252495), the most reported AE was mild headache. No clinically relevant changes were observed in vital signs, body weight, laboratory variables, and ECG findings. A greater reduction in hemoglobin, hematocrit, and RBC count was observed in patients with hepatic impairment [26]. In another study by Schlaich et al. (NCT03541174), the most frequent AE was edema or fluid retention, primarily occurring in the first four weeks of treatment. This AE was reported to occur more frequently with ACT than with placebo in a dose-dependent manner. Both edema and fluid retention were generally mild or moderate, and it was reported to be more common in patients with chronic kidney disease. A reversible decrease in hemoglobin concentrations was also noted. In contrast to the study mentioned above, a moderate increase in body weight was observed with both doses. The study also demonstrated a 5-mmHg reduction in office SBP, which was associated with a 10% relative risk reduction in major cardiovascular events, which is particularly relevant for patients with resistant HT who are at high risk for CVD. The withdrawal phase confirmed the sustained BP-lowering efficacy of ACT. Some participants experienced loss of renal function, though this was not believed to be associated with ACT, but rather their preexisting conditions. ACT was also found to have an antiproteinuric effect, which appeared more pronounced in patients with chronic kidney disease, suggesting a potential role in reducing organ damage [25].

A study by Verweij et al. emphasized that ACT’s long half-life is advantageous as it should maintain a decrease in BP following a missed dose. The study also noted that BP reductions were greater in white patients (*p* = 0.0084 and *p* = 0.037, for SBP and DBP, respectively), while no statistically significant differences were observed between sexes. Furthermore, there were no clinically relevant differences in the AEs between the ACT and control groups. The most common AE here was also fluid retention [18]. No studies have indicated clinically relevant hepatotoxicity associated with ACT [17].

All in all, all studies found that the plasma concentration of ACT declined slowly, that there were no severe AEs, and that it was well tolerated across all doses, indicating a promising potential for ACT.

### 5.2. Aprocitentan vs. Standard Treatment

ACT is a relatively new drug, making it important to clarify that only a limited number of studies have been conducted. HT is one of the most common disorders [29], yet, as previously mentioned, only 42% of adults with HT are properly diagnosed and receive appropriate treatment, and only 1 in 5 have it under control (2). This clearly indicates that there is substantial room for improvement. Additionally, approximately 10–20% of patients with HT have resistant HT [30].

Pharmacotherapy for resistant HT offers limited effective options, and yet the ET-1 pathway has been overlooked in treatment, making ACT unopposed in targeting this pathobiological pathway [29,31]. Several studies have found that ACT significantly reduces systolic and diastolic BP at doses of 10 and 25 mg [29]. Furthermore, ACT is the only ERA under development for resistant HT, indicating its relevance and ability to contribute to standard treatment. Moreover, it has shown both additive and synergistic effects when combined with other antihypertensive agents, such as valsartan and enalapril, emphasizing the unique potential when combined with the RAAS system [32]. ACT was deemed well tolerated over all doses and has shown potential organ-protective effects in patients with kidney disease [25,32]. While inhibition of the RAAS remains a fundamental and non-substitutable component in the management of hypertension and heart failure, largely owing to its well-documented antiproliferative and anti-inflammatory properties, the PRECISION trial data suggest that targeting an alternative pathway, compared to the standard treatment, such as the ET-1 pathway, may be more beneficial than merely combining classical antihypertensive drugs in patients with resistant HT [25,30].

However, it should be considered, that these results, encouraging as they are, were obtained on the basis of relatively small trials with short follow-up periods. To fully prove its potential, ACT needs to be further analyzed in large multi-center trials with longer follow-up times so that its long-term efficacy can be further explored and potential rare adverse effects can be detected.

## 6. Materials and Methods

**Figure 2:**
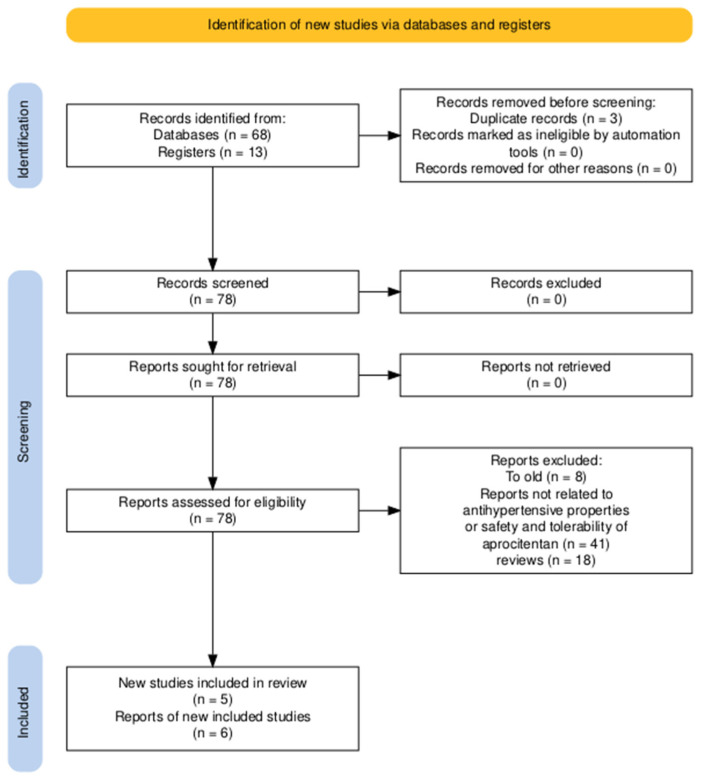
Prisma flow diagram (https://estech.shinyapps.io/prisma_flowdiagram/, last accessed on 17 May 2025 [33]).

The literature in this thesis was found through online databases and a few individual web pages. Online databases included PubMed (https://pubmed.ncbi.nlm.nih.gov/?otool=idkdnlblib, last accessed on 17 May 2025) and Clinical trials (https://clinicaltrials.gov/, last accessed on 17 May 2025). Primary literature was found by a systematic literature search on PubMed, through written reviews and meta-analyses. Searches were, if possible, restricted to a period from 2018 to 2025, to ensure the most recent data. The search terms was: (((((Hypertension[Title/Abstract]) OR (arterial hypertension[Title/Abstract])) OR (“high blood pressure”[Title/Abstract])) OR (“cardiovascular disease”[Title/Abstract])) OR (“Hypertension”[Mesh])) AND (Aprocitentan), or aprocitentan in itself. Clinical trials were included in this thesis to assess the efficacy and safety of aprocitentan. The search process is outlined in the PRISMA flow diagram, Figure 2 [33].

## 7. Conclusions

HT is a global disease that is frequently associated with comorbidities. It is becoming increasingly prevalent and represents a growing burden on public health. Consequently, resistant HT is also likely to become more prevalent. Studies conducted in both humans and rats have demonstrated that ACT significantly reduces BP in individuals with resistant HT, as well as works well with other anti-hypertensive agents. Additionally, no severe AEs were reported in the available studies. Moreover, ACT targets a distinct pharmacological pathway compared to existing antihypertensive agents, supporting its potential as a valuable therapeutic option for resistant HT. However, ACT is still a relatively new drug, and only a limited number of studies have been conducted to date. In addition, the studies have their limitations, such as relatively small study populations, which decreases generalization. Furthermore, none of the studies investigate the long-term AEs of ACT. Therefore, further research involving a larger study population and extended trial durations is needed to fully evaluate its efficacy and safety.

## Figures and Tables

**Table 1:** Overview of plasma pharmacokinetics of aprocitentan.

Parameter	5 mg ACT	25 mg ACT	100 mg ACT	100 mg ACT (Elderly)
C_max_ (μg/mL)	0.83	3.57	13.41	17.62
t_max_ (h)	4.00	4.50	7.00	6.00
t_1/2_ (h)	48.83	45.69	41.69	44.71

Values are expressed as medians. C_max_ = maximum plasma concentration. t_max_ = time to reach maximum plasma concentration. t_1/2_ = terminal half-life. The table is based on information from Sidharta et al. [19].

**Table 2:** Overview of completed clinical trials using aprocitentan in relation to HT.

Title	Design	Results	Conclusion
Study to evaluate how aprocitentan is safe and how it is absorbed and broken down in the body of Japanese and Caucasian subjects NCT03586570 [20]	Single-center, double-blinded, placebo-controlled, randomized study. Phase 1. 20 participants, 10 Japanese and 10 Caucasian	C_max_ = 3.5 h, t_½_ = 49.1 and 48.8 h for Japanese and Caucasian, respectively. Steady-state conditions were reached within 7–10 days. A greater decrease in mean and median hemoglobin, hematocrit and RBC, was observed, but was not considered clinically relevant	No clinical difference in exposure, safety, and tolerability to aprocitentan on the two ethnic groups were found. The study deemed it unlikely that the PK of aprocitentan would differ significantly between Caucasians and other ethnicities
Phase 3 PRECISION study NCT03541174 [25]	Multi-center, blinded, randomized, parallel-group, phase 3 trial The study consisted of 3 sequential parts: part 1 was a 4-week double-blinded, randomized and placebo-controlled part. Part 2 was a 32-week single-blinded part. Part 3 was a 12-week double-blinded randomized, and placebo-controlled withdrawal part. 1965 individuals were screened, and 730 participants were randomly assigned.	Part 1 showed the least square mean change in office SBP was −15.3 mmHg for ACT 12.5 mg, −15.2 mmHg for ACT 25 mg and −11.5 mmHg for placebo. DBP also decreased for both doses, when compared to placebo (−3.9 mmHg and −4.5 mmHg, respectively). It was maintained for patients. Receiving ACT and decreased in patients previously receiving placebo, during part 2. A significant increase in BP was seen with placebo compared to ACT	Aprocitentan was found efficient to lower BP. Additionally, it was found to provide clinically meaningful lowering of SBP and DBP in patients with resistant HT with manageable adverse effects
Single-dose pharmacokinetics, safety, and tolerability of the dual endothelin receptor antagonist aprocitentan in subjects with moderate hepatic impairment NCT04252495 [26]	Open-label, phase 1 study 17 participants: subjects were 8 subjects with moderate hepatic impairment matched with 9 healthy subjects 1 subject was excluded due to personal reasons = 16 subjects completed	C_max_ was reached at a median of 4 h for both groups. Concentration decreased slowly in both the subjects with moderate hepatic impairment and the healthy subjects, indicated by a t_½_ of 56.4 h and 48.3 h, respectively	Aprocitentan was found to be absorbed similarly for moderate hepatic impaired compared to healthy subjects. No clinically relevant difference in PK, safety and tolerability was found. Aprocitentan can be administered to subjects with mild and moderate hepatic impairment, without dose adjustment.
Dose-finding study with ACT-132577 in participants with essential hypertension NCT02603809 [18]	Multi-center, double-blind, double-dummy, randomized, placebo- and active-reference, parallel group, phase 2, dose-finding study. 490 participants; 430 completed	Clinically relevant decrease in BP occurred, within 2 weeks in the aprocitentan 10, 25 and 50 mg groups. A statistically significant dose–response relationship for the change in mean siDBP was found	Aprocitentan 10, 25, and 50 mg once daily lowered BP in a clinically relevant, dose dependent manner. The maximum effect on BP was observed at 25 mg.

ACT: aprocitentan, ABPM: ambulatory blood pressure measurement, BP: blood pressure, C_max_: maximum plasma concentration, t_½_: terminal half-life, DBP: diastolic blood pressure, PK: pharmacokinetics, SBP: systolic blood pressure, siDBP: mean sitting diastolic blood pressure.

## Data Availability

No new data were created or analyzed in this study. Data sharing is not applicable to this article.

## Abbreviations

The following abbreviations are used in this manuscript:

ACE	Angiotensin converting enzyme
ACEI	ACE inhibitor
ACT	Aprocitentan
AE	Adverse effect
Ang I	Angiotensin I
Ang II	Angiotensin II
ARBs	Angiotensin receptor blockers
BARB	Beta adrenergic receptor-blockers
BCRP	Breast cancer resistance protein
BP	Blood pressure
CCB	Calcium channel blockers
CO	Cardiac output
CVD	Cardiovascular disease
DBP	Diastolic blood pressure
DOCA	Deoxycorticosterone acetate
ERA	Endothelin receptor antagonist
HBPM	Home blood pressure measurements
HR	Heart rate
HT	Hypertension
MAP	Mean arterial pressure
NO	Nitric oxide
RAAS	Renin–angiotensin–aldosterone system
SBP	Systolic blood pressure
SHRs	Spontaneously hypertensive rats
SV	Stroke volume
TPR	Total peripheral resistance
WHO	World Health Organization
